# Association of trace metal elements with lipid profiles in type 2 diabetes mellitus patients: a cross sectional study

**DOI:** 10.1186/s12902-017-0217-z

**Published:** 2017-10-13

**Authors:** Amare Desalegn Wolide, Belay Zawdie, Tilahun Alemayehu, Samuel Tadesse

**Affiliations:** 1 Department of Medical Physiology, College of Health Sciences, Jimma University, 378 Jimma, Ethiopia; 2 Department of Medical Biochemistry, College of Health Sciences, Jimma University, 378 Jimma, Ethiopia; 3 Department of Human Anatomy, College of Health Sciences, Jimma University, 378 Jimma, Ethiopia

**Keywords:** Type 2 diabetes mellitus, Dyslipidemia, Trace metal elements

## Abstract

**Background:**

It is well known that dyslipidemia and chronic hyperglycemia increase the onset of diabetes and diabetic complication. The aim of this study is to see the association of trace metals elements and lipid profile among type 2 diabetes mellitus patients.

**Methods:**

The study was conducted on 214 type 2 diabetic patients at Jimma University Specialized Hospital, Jimma, Ethiopia. All the eligible study participants responded to the structured interviewer administered questionnaire and fasting venous blood was drawn for biochemical analysis. Trace metal elements (zinc(Zn^+2^), magnesium(Mg^+2^), chromium(Cr^+3^), calcium(Ca^+2^), phosphorus(Po_4_^−3^), manganese(Mn^+2^), copper(Cu^+2^), and iron(Fe^+3^)) and lipid profiles (total cholesterol (TC), low-density lipoprotein cholesterol (LDL-C), high-density lipoprotein (HDL-C), and triglycerides (TG)) were measured by atomic absorption spectrophotometry and enzymatic determination method respectively. Data were analyzed by SPSS version 24 software for windows. Bonferroni correction for multiple statistical comparisons was used and a *p*-value less than 0.01 were accepted as a level of significance.

**Result:**

The mean age of study participants was 42.95(±12.6) with an average of 5.83(±3.1) years being diagnosed with diabetes mellitus. The BMI of female (27.1(±4.9)) was significantly higher than male (25.21(±4.2)). BMI shows positive and significant (*p* < 0.01) association with lipid profiles (TC, LDL-C, and TG) among type 2 diabetic patients in the liner regression model. In addition, WH-R was positively associated with TG. In Pearson partial correlation adjusted for sex and age, Za^+2^ shown to have statistically significant and negative correlations with TC, LDL-C and with TG. Mg^+2^ and Cr^+2^ negatively and significantly correlated with the lipid profile TC and LDL-C. Ca^+2^ negatively correlated with TC and TG. Po^−3^_4_ positively correlated with HDL-C; iron negatively correlated with TC. However, in the liner regression model, only calcium positively and significantly (Beta = −0.21, *p* < 0.01) associated with TG.

**Conclusion:**

In the current study, a negative correlation was observed between trace metal elements (Zn^+2^, Mg^+2^, Cr^+3^, Ca^+2^ and Fe^+3^) and lipid profile (TC, LDL-C and TG) among type 2 diabetes mellitus patients. In addition, Ca^+2^ observed to be associated with TG. Future studies are highly advised to uncover the bidirectional association between trace metal element and dyslipidemia in diabetic patients.

## Background

It is a scientific fact that dyslipidemia and chronic hyperglycemia increase the onset of diabetes and diabetic complication [1, 2]. Now, the world has seen an unexpected rise in the prevalence of diabetes [3]. The prevalence of people with type 2 diabetes will increase from 350 million today to 592 million by 2035 [4, 5]. Dyslipidemia is the major characteristic of almost to all type 2 diabetic patients and the underlying mechanism is not resolved well [6]. Diabetic dyslipidemia is characterized by the presences of a high proportion of very low-density lipoprotein (VLDL-C), higher fasting and postprandial triglycerides (TG), elevated low-density lipoprotein cholesterol (LDL-C) and low high-density lipoprotein cholesterol (HDL-C) [7]. Diabetic patients often have seen impaired metabolism of trace metal elements [8]. The exact mechanism between diabetes and impaired trace metal elements hemostasis is clearly unknown [9–11]. Trace metal elements can affect the synthesis, secretion, release, and mechanism of action of insulin [12–14]. The study aim was to determine the association between trace metal elements and the lipid profiles of type 2 diabetes mellitus (T2DM) patients at Jimma University Specialized Hospital, Ethiopia. To the best knowledge of the researchers, this study is the first of its kind in Ethiopia. Furthermore, we do believe that it will serve as a baseline for future research in the area.

## Methods

### Study subjects and period

A cross-sectional study was conducted in the outpatient department of Jimma University Specialized Hospital (JUSH) from February 15, 2015-October 30, 2015. Informed consent was obtained from 214 type 2 diabetes mellitus patients. Patients who are pregnant or lactating; using any drugs affecting electrolytes, taking nutritional supplements, such as magnesium-containing laxatives; suffering from chronic disorders of the liver, kidney and cardiovascular system, endocrine disorders, established psychiatric disorder and on antidepressant and /or antipsychotic therapy, HIV/AIDS, malignancy and, substance abuse were excluded from the study. The study participants were selected by consecutive sampling technique.

### Anthropometric measurement

Anthropometric data such as weight (Kg) and height was determined to the nearest 0.1 kg and 0.1 cm respectively. Body mass index (BMI) was calculated by dividing weight (Kg) by the square of height in meter (m^2^). Waist and hip circumference were measured in centimeters and eventually, a waist-hip ratio was calculated.

### Clinical and blood parameters measurement

Structured interviewer administered questionnaire was used to collect data on demographics, duration of diabetes, family history of diabetes mellitus and other chronic diseases. Both systolic and diastolic blood pressure was measured by mercury sphygmomanometer two times and the average values were computed. A 5 ml overnight venous blood sample was collected from each study subjects. Collected blood samples were stored in ethylenediamine tetraacetic acid potassium salt (EDTA.K3)-containing vacuum tubes (Weihai Hongye, Weihai, China) and plain vacutainer tubes (Shandong Branden Medical Devices Co., Ltd., Beijing, China). Blood sample in the EDTA K3 containing tube used for Fasting blood glucose (FBG) determination. Blood in the plain vacutainer tube was allow clotting and centrifuged at 3000 rpm for 10 min to separate the serum from the whole blood for lipid profile and Trace metal element determination.

### Specimen preparation and analysis

Fasting plasma glucose (FBG) and serum TC, HDL-C, LDL-C, and TG were determined by glucose hexokinase and by enzymatic method. Both clinical methods were performed using automated chemistry analyzer ABX Pentra 400 (HORIBA Medical Diagnostics Instruments & Systems, Montpellier, France) [15]. Serum trace metal elements such as zinc (Zn^+2^), magnesium (Mg^+2^), chromium (Cr^+3^), calcium (Ca^+2^), phosphorus (Po_4_^_3^), manganese (Mn^+2^), copper (Cu^+2^), and iron (Fe^+3^) were determined using wet acid digestion method. All the sample containers were washed and rinsed with distilled water and dried in the oven at 80 °C for 24 h. Then, the serum in each container mixed with 3 mL concentrated nitric acid and hydrogen peroxide [HNO3–H2O2] solution (2:1, *v*/v) for 10 min. Then, the beaker was covered by 150 mm pyrex watch glass and allowed to be heated on a hot plate until complete digestion had taken place at 120 °C. After the solution has cooled, the digested samples were centrifuged at 10,000 rpm for 30 s. Then, the organic supernatant aspirated directly into the flam and the sample was analyzed by atomic absorption spectrophotometry (Shimadzu AA-670, Kyoto, Japan) as described in our previous publications [16].

### Data quality assurance

Pretest before the actual dates of data collection and subsequent revisions during and after data collections days has been done to ensure the quality of the data. Experienced and trained data collectors were recruited for Scio demographic, clinical and laboratory data collections. Careful attention was given from the beginning of blood withdrawal until analysis. All reagents, controls, and calibrators used were checked for their expiry date and used according to the manufacturer’s instructions.

### Ethics approval and consent to participate

The study was approved by the institutional review board of Jimma University, college of health sciences, Ethiopia with the reference number RPGC/4010/2015. The research has been conducted according to the declaration of Helsinki for medical research. Consent for participation in the study was obtained from study participant voluntarily after having a clear understanding of all the purpose of the study.

### Data analysis

Statistical Package for the Social Sciences (SPSS) software version 24 (SPSS Inc., Chicago, IL, USA) was used to analyze data. Descriptive statistics, Pearson partial correlation test and linear regression analysis were compute to addresses the research questions. All the assumption of the model was satisfied. Moreover, adjustment for multiple tests (Bonferroni adjustments) was used [16–19] and a *p*-value less than 0.01 was set as level of significant.

## Results

Initially, a total of 239 type 2 diabetes mellitus patients were recruited. However, 214 patients were eligible for the study. The mean age of diabetes patients, diabetes durations, BMI, WH-R SBP and DBP were 42.95 ± 12.64, 5.83 ± 3.11, 26.15 ± 4.53, 1.45 ± 0.56, 134.2 ± 22.62, and 88.36 ± 13.23 respectively. Female type 2 diabetic patients showed significantly higher mean BMI as compared to male type 2 diabetes patients. However, a significant mean difference was not detected among male and female type 2 diabetic patients with respect to age, diabetes duration, WH-R, SBP, and DBP. Based on the International Diabetic federation (IDF) cut of points [20], majority of the study participants 132.1(61.7%), 169(79.0%), and 115(53.7%) had high undesirable BMI, WH-R, and SBP (Table 1).

**Table 1:** Descriptive statistics (mean ± standard deviations and frequency) on the age, diabetes duration, BMI, WH-R, SBP and DBP of type 2 diabetes patients

Study parameters		Sex		Total *N* = 214(100%)
		Male *N* = 107(50%)	Female *N* = 107(50%)	
Age (years)	Mean (± Std.)^a^	41.89(±13.5)	44.02(±11.6)	42.95(±12.6)
Diabetes duration (years)	Mean (± Std.)^a^	5.66(±3.1)	5.99(±3.2)	5.83(±3.1)
BMI (Kg/m2)	Mean (± Std.)^a^	25.21(±4.2)	27.1(±4.9)**	26.1(±4.5)
	Obese	17(7.9%)	638(17.8%)	55(25.7%)
	Over Wight	43(20.1%)	34(15.9%)	77 (36.0%)
	Normal	40(18.7)	30(14.0%)	70(32.7%)
	Under Wight	7(3.3%)	5(2.3%)	12 (3.3%)
WH-R (cm)	Mean (± Std.)^a^	1.4(±0.6)	1.5(±0.6)	1.4(±0.6)
	High	85(39.7%)	84(39.3%)	169(79.0%)
	Low	22(10.3%)	23(10.7%)	45(21.0%)
SBP (mmHg)	Mean (± Std.)^a^	131.8(±23.4)	136.5(±21.6)	134.2(±22.6)
	High	53(24.8%)	62(29.0%)	115(53.7%)
	Low	54(25.2%)	45(21.1%)	99(46.3%)
DBP (mmHg)	Mean (± Std.)^a^	87.5(±13.2)	89.3(±13.3)	88.3(±13.2)
	High	53(24.8%)	62(29.0%)	115(53.7%)
	Low	54(25.2%)	45(21.0%)	99(46.3%)

Key:

^a^Independent t- test

**Significant mean difference (*p* < 0.01)

In the linear regression model, age, diabetes duration, and WH-R (except for TG) are not associated significantly with the lipid profiles (TC, HDL-C, LDL-C, and TG) of the patients. However, BMI significantly (*p* < 0.001) and positively associated with patients’ serum TC, LDL-C, and TG (Table 2).

**Table 2:** liner regression analysis between age, diabetes duration, BMI and WH-R with the lipid profiles of type 2 diabetes patients

Study parameters	Coefficients^a^		Coefficients^b^		Coefficients^c^		Coefficients^d^	
	Beta	*p*-value	Beta	*p*-value	Beta	*p*-value	Beta	*p*-value
Age (years)	0.04	0.54	−0.11	0.16	0.06	0.35	−0.01	0.89
Diabetes duration (years)	−0.014	0.86	0.10	0.19	−0.03	0.62	0.03	0.64
BMI (Kg/m2)	0.65	<0.001*	−0.38	<0.001*	0.64	<0.001*	0.43	<0.001*
WH-R(cm)	0.04	0.48	0.15	0.07	−0.11	0.13	0.23	<0.001*

Key:

Coefficients ^a^Dependent Variable: TC (mg/dL)

Coefficients ^b^Dependent Variable: HDL-C (mg/dL)

Coefficients ^c^Dependent Variables: LDL-C (mg/dl)

Coefficients ^d^Dependent Variables TG (mg/dL)

Beta: Standardized Coefficients

*Statistical significant at *p*-value <0.01

To investigate the relationship between detected trace metal elements and lipid profile in the serum of type 2 diabetic patients, the partial correlation analysis adjusted to age and sex was applied. Thus, Zn^+2^ was negatively correlated with TC, LDL-C and TG. In the same manner, both Mg^+2^ and Cr^+3^ negatively correlated with TC and LDL-C, but not with TG. Ca^+2^ were correlated negatively with TC and TG. Furthermore, Fe^+3^ were negatively correlated with TC (Table 3).

**Table 3:** Pearson partial correlation analysis between lipid profile and trace metal elements among type 2 diabetes study subjects adjusted for sex and age

Study parameters control for age and sex		TC (mg/dL)	HDL-C (mg/dL)	LDL-C (mg/dL)	TG (mg/dL)
Zn^+2^ (μg/dL)	Correlation	−0.269	0.218	−0.261	−0.245
	Significance (2-tailed)	<0.001*	<0.001*	<0.001*	<0.001*
Mg^+2^ (mg/dL)	Correlation	−0.200	0.284	−0.237	−0.151
	Significance (2-tailed)	0.003*	<0.001*	<0.001*	0.028
Cr^+3^ (μg/L)	Correlation	−0.173	0.226	−0.204	−0.096
	Significance (2-tailed)	0.012*	<0.001*	0.003*	0.162
Ca ^+2^ (mg/dL)	Correlation	−0.176	0.140	−0.103	−0.225
	Significance (2-tailed)	<0.01*	0.041	0.133	<0.001*
Po_4_^_3^ (mg/dL)	Correlation	−0.142	0.195	−0.120	−0.117
	Significance (2-tailed)	0.039	0.004*	0.080	0.090
Mn^+2^ (μg/L)	Correlation	−0.091	0.023	−0.117	−0.017
	Significance (2-tailed)	0.189	0.742	0.088	0.809
Cu^+2^ (μg/dL)	Correlation	−0.031	0.021	−0.077	0.096
	Significance (2-tailed)	0.658	0.761	0.264	0.165
Fe^+3^ (μg/dL)	Correlation	−0.186	−0.005	−0.162	−0.092
	Significance (2-tailed)	0.007*	0.940	0.020	0.183

*Correlation is significant at *p* < 0.01 (2-tailed)

Finally, liner regression enter model showed negative and significant associated between Ca^+2^ and TG (Beta = −0.21, *p* < 0.01) (Table 4).

**Table 4:** liner regression analysis between trace metal elements and lipid profiles

Study parameters	Coefficients^a^		Coefficients^b^		Coefficients^c^		Coefficients^d^	
	Beta	*P*-value	Beta	*P*-value	Beta	*p*-value	Beta	*p*-value
Zn^+2^ (μg/dL)	−0.25	0.02	0.05	0.65	−0.21	0.05	−0.23	0.03
Mg^+2^ (mg/dL)	0.01	0.98	0.20	0.06	−0.06	0.55	0.05	0.63
Cr^+3^(μg/L)	−0.06	0.38	0.10	0.17	−0.10	0.180	−0.01	0.88
Ca^+2^ (mg/dL)	−0.14	0.07	0.06	0.46	−0.06	0.41	−0.21	0.01*
Po_4_^−3^ (mg/dL)	−0.08	0.34	0.12	0.13	−0.09	0.33	−0.02	0.82
Mn^+2^ (μg/L)	−0.07	0.32	−0.01	0.89	−0.07	0.32	−0.04	0.53
Cu^+2^ (μg/dL)	−0.05	0.51	0.07	0.34	−0.09	0.22	0.06	0.43
Fe^+3^ (μg/dL)	−0.17	0.02	0.01	0.85	−0.14	0.04	−0.09	0.21

Key:

Coefficients ^a^Dependent Variable: TC (mg/dL)

Coefficients ^b^Dependent Variable: HDL-C (mg/dL)

Coefficients ^c^Dependent Variables: LDL-C (mg/dl)

Coefficients ^d^Dependent Variables TG (mg/dL)

Beta: Standardized Coefficients

*Statistical significant at *p*-value <0.01

## Discussion

Diabetes is a chronic metabolic disease affecting the whole world. Especially peoples living in the developing part of the world are becoming more victim of diabetes because of globalization; urbanization and sedentary life. Generally, research findings in the past revealed the strong associations between disturbed blood parameters and end glycated products in the blood sample of diabetes patients. For example, findings were reported an abnormal relationship between plasma trace metal element amount and hyperglycemia in the blood sample of diabetic patients. However, osmotic dieresis due to hyperglycemia has been mentioned as a potential factor for the presences of disturbed trace metal elements in the blood sample of diabetic patients [21]. In the same extent, the complexity of diabetic dyslipidemia is still a question for the researchers. However, low level of insulin, reduced activity of lipoprotein lipase enzyme, increased activities of proprotein convertase subtilisin/kexin 9 (PCSK9) and cholesteryl ester transfer protein (CETP) plus impaired clearance of lipoproteins was identified as aggravating factors involved in the pathophysiology of diabetic dyslipidemia [1, 6, 22–24]. According to the finding of this particular study, BMI was positively and significantly associated with serum TC, LDL-C, and TG. Thus, this finding confirms that BMI still can predict the patients’ lipid level in the blood and considered as an alternative method to determine blood lipid levels. In addition, the current study also demonstrates the significant difference of BMI in both genders of type 2 diabetic patients. Female type 2 diabetic patients had significantly higher BMI (27.1(±4.9)) than male (25.21(±4.2)). The finding was consistent with the report of the study conducted in Low-Income Muslim Uyghur Women in Far West China [25]. The higher percentages of body fat in women could be due to the lipogenic sex hormone called estrogen [26]. Thus, being biologically female could increase BMI and lipid accumulation in the blood and more vulnerable to metabolic syndrome [27]. In the current study, Zn^+2^ were the only trace metal element strongly shows significant negative correlation with TC, LDL-C, TG and positive significant correlation with HDL-C. It is a mineral that plays a vital role in many biological processes and plays an important role in the action of insulin and carbohydrate metabolism [28]. Daily intake and supplementation of Zn^+2^ have been associated with low blood cholesterol level and reduction from any form of metabolic risks (diabetes, heart disease, and hyper-triglyceridemia) [29, 30]. The negative relationship of Zn^+2^ with TC, LDL-C, and TG among type 2 diabetic patients, in the current study, is in harmony with other clinical trial and intervention studies [31–33]. Some other studies showed that no change has been observed in the blood cholesterol level of diabetic patients despite Zn + 2 supplementation [34–36]. The disagreement of the findings might be due to difference in study designs, diabetic complications, glycemic control, and the behavioral and Socio-cultural difference. Like other trace metal elements, the adequate amount of Mg^+2^ and Cr^+3^in the blood would play a greater role in controlling metabolic crisis among diabetes mellitus patients [37–40]. Mg^+2^ plays an extremely important role in the activation and modulation of many enzymes that are involved in carbohydrate and insulin metabolism. Furthermore, it is playing a crucial role in the metabolism of lipid peroxidation since it acts as a cofactor in the cholesterol synthesis and 3-Hydroxy-3-methyl-glutaryl-CoA (HMG-CoA) reductase enzyme [41]. The inverse relationship of Mg^+2^ with TC and LDL-C in the current study is in agreement with the study amid to assess the correlation between Mg^+2^ and glycemic control and lipid profile among children with type 1 diabetes and adult patients with type 2 diabetes [38, 42]. Moreover, both human and animal experimental studies showed a significant reduction of TC, LDL-C,TG and increase the level of HDL-C after Mg^+2^ supplementation [43–46]. However, a 12-week randomized double-blind study revealed no significant inverse relationships of Mg^+2^ and lipid profiles [47]. The trivalent metal element Cr^+3^ enhances the action of insulin in the uptake of glucose at the cellular level [48]. Cr^+3^ serves as an important antioxidant element that improves glucose intolerance and dyslipidemia [49, 50]. According to our founding, Cr^+3^ negatively correlated with TC and LDL-C. In addition, Cr^+3^ positively correlated with HDL-C. This funding got similarity with the study where Cr^+3^ supplementation showed to improve HDL-C and reduce the TC level among patients with diabetes as it is stated in the review paper [39]. Ca^+2^ is a chief divalent metal element that would play a key role in the muscle contraction, nerve excitability, blood coagulation, and secondary messenger system. In addition, it is also involved in the bone and tooth physiology. The blood Ca^+2^ level should be regulated in a narrow range otherwise; it may cause undesirable physiological changes immediately. Impaired insulin secretion and action in the peripheral cells might be associated with low calcium concentration [51, 52]. Hypocalcemia could reduce the secretion of insulin and by doing so; it may increase the accumulations of lipids in the body. In this study, serum calcium inversely correlated with TC and TG. Moreover, Ca^+2^ negatively predicted the serum concentration of TG level in the linear regression model. Lastly, Fe^+3^ showed a negative correlation with TC. Iron plays an essential role as a cofactor for fuel oxidation and electron transport, but it also has the potential to cause oxidative damage if not carefully regulated [53]. Iron accumulation in the blood used as a biomarker for diabetes complication [54–56]. Our earlier work [53] shows that the mean level of iron in type 2 diabetes patients was low as compared with normal healthy individuals. It actually deviates from the fact of many findings done on the relationship between diabetes and iron concentration in the blood. Nevertheless, the low blood iron concentration and its negative correlation with TC could be because our study subjects might have exposed to intestinal parasitic infection or lack of access to iron rich foods.

### Limitation of the study

Because of the limited fund, we had, postprandial glucose, insulin, and glycated hemoglobin level of patients not measured. Selection bias might be there since study participants were selected by consecutive sampling technique. Moreover, the study was cross-sectional and the relationship between the measured parameters may not be truly associated.

## Conclusion

Our study shows positive and negative correlation and association of trace metal elements with the lipid profile of the T2DM patients.

## Abbreviations

BMI: Body mass index
Calcium: Ca^+2^
Chromium: Cr^+3^
Copper: Cu^+2^
DBP: Diastolic blood pressure
DM: Diabetes Mellitus
HLD-C: High-density lipoprotein cholesterol
Iron: Fe^+3^
JUSH: Jimma University Specialized Hospital
LDL-C: Low-density lipoprotein cholesterol
Magnesium: Mg^+2^
Manganese: Mn^+2^
Phosphors: Po_4_^_3^
SBP: Systolic blood pressure
SPSS: Statistical Package for the Social Sciences
T2DM: Type 2 Diabetes Mellitus
TG: Triglycerides,
VLDL-C: Very low-density lipoprotein
WH-ratio: Waist-hip ratio
Zinc: Zn^+2^
